# Antioxidant and Immunoregulatory Activity of Polysaccharides from Quinoa (*Chenopodium quinoa* Willd.)

**DOI:** 10.3390/ijms151019307

**Published:** 2014-10-23

**Authors:** Yang Yao, Zhenxing Shi, Guixing Ren

**Affiliations:** Institute of Crop Science, Chinese Academy of Agricultural Sciences, Beijing 100081, China; E-Mails: yaoyang@caas.cn (Y.Y.); shizhenxingmu@gmail.com (Z.S.)

**Keywords:** quinoa, water-extractable polysaccharide, alkali-extractable polysaccharide, bioactivity

## Abstract

The water-extractable (QWP) and the alkali-extractable (QAP) polysaccharides from quinoa (named QWP and QAP, respectively) and their four polysaccharide sub-fractions (QWP-1, QWP-2, QAP-1 and QAP-2), were isolated and purified by anion-exchange and gel filtration chromatography. QWP-1 and QWP-2 were composed of Rha, Ara, Gal and GalA. QAP-1 and QAP-2 were composed of Rha, Ara, Man, Gal and GalA. Antioxidant and immunoregulatory activities of the polysaccharides were evaluated. The results showed that QWP-1, QWP-2, QAP-1 and QAP-2 had significant antioxidant and immunoregulatory activities. The results suggest that QWP-1, QWP-2, QAP-1 and QAP-2 could be used as potential antioxidants and immunomodulators.

## 1. Introduction

Currently, polysaccharides from natural sources have attracted increased attention due to their potential biological functions, especially antioxidant and immunomodulation activities such as scavenging free radicals, inhibiting lipid oxidation, promoting natural killer cells (NK) cytotoxicity, and activating macrophages and interleukins [1,2,3,4]. The bioactivity of polysaccharides mainly depends on several structural parameters including sugar composition, molecular weight, type of glycosidic bond, and degree of sulfation [5]. In view of their potential application in functional foods and medicine, more and more studies have focused on the isolation and purification of polysaccharides from many plants, animals and microorganisms [6].

Quinoa (*Chenopodium quinoa* Willd.) is a well-known staple food of the Andean zone; it is a pseudo-cereal used principally in the same manner as wheat and rice [7]. Quinoa seeds contain carbohydrates, high-quality protein, a balanced amino acid spectrum of high lysine and methionine contents, and are rich in dietary fiber [8]. There is now much interest in quinoa for its physiological functionalities, such as antimicrobial [9], anti-inflammatory [10] and cholesterol lowering activities [11].

Most of the reported phytochemicals in terms of quinoa are saponins and phenolic acids [12]. However, polysaccharides have scarcely been reported [13]. In this work, we isolated polysaccharides from quinoa, identified their chemical characteristics, and determined the antioxidant and immunomodulatory effects. The aim of this study was to better understand structural characteristics, antioxidant and immunostimulating activity of polysaccharides from the seeds of quinoa. They were separated and purified simultaneously by 2-diethylaminoethyl cellulose (DEAE) Sepharose Fast Flow anion-exchange chromatography and Sephacryl S-300 High Resolution gel chromatography. Furthermore, we evaluated *in vitro* the antioxidant (1,1-diphenyl-2-picrylgydrazyl (DPPH) assay and Oxygen Radical Absorption Capacity (ORAC) assay) and immunomodulation activity (nitric oxide release, and cytokine secretion).

## 2. Results and Discussion

### 2.1. Extraction and Purification of Polysaccharide

**Figure 1:**
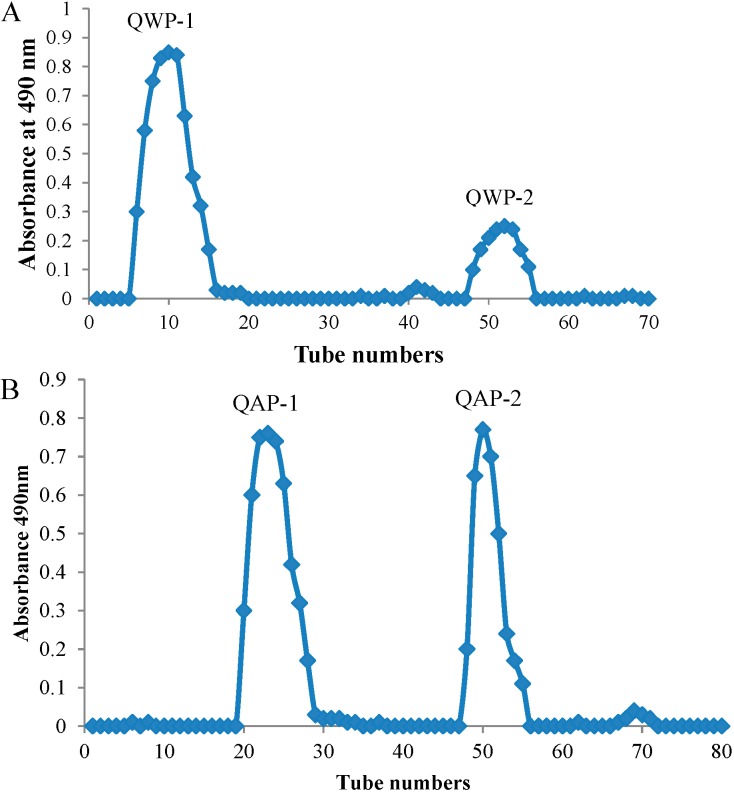
Elution curve of the water-extractable (QWP) and the alkali-extractable (QAP) on a Sepharose Flast Flow column. (**A**) QWP and (**B**) QAP. The crude polysaccharide was dissolved in distilled water and applied to the column, elution with distilled water and NaCl (0.0–2.0 M). The elution solution was collected and the carbohydrate content of the collected fraction was monitored by phenol–sulfuric acid method.

The water-extractable (QWP) and the alkali-extractable (QAP) were isolated from the seeds of quinoa and the yield was approximately 5.35% and 2.55%, respectively. The purification was conducted on a DEAE Sepharose Fast Flow column to obtain water-eluted and salt-eluted fractions, accounting for 42.3%, 22.8%, 48.2% and 15.1% of QWP-1, QWP-2, QAP-1 and QAP-2 by weight, respectively (Figure 1). Based on the molecular weight difference, the two fractions were then further purified using Sephacryl S-300 column.

### 2.2. Molecular Weight and Monosaccharide Composition

The high-performance size-exclusion chromatography with refraction-index detection (HPSEC-RID) elution profile showed mainly single and symmetrically sharp peaks which reveal 100.00%, 97.25%, 96.25% and 99.72%, of the QWP-1, QWP-2, QAP-1 and QAP-2 homogeneous polysaccharides, respectively. HPSEC-MALLS analysis revealed the *M*_w_ (molecular weight average) of QWP-1, QWP-2, QAP-1 and QAP-2 as 26, 37, 34 and 22 kDa, respectively.

Analysis of Gas chromatography (GC) indicated that QWP-1 was composed of Rha, Ara, Gal and GalA in a molar ratio of 2.03:1.34:0.68:3.27. QWP-2 was consisted of Rha, Ara, Gal and GalA with a relative molar ratio of 1.52:0.18:4.67:0.97. QAP-1 was composed of Rha, Ara, Man, Gal and GalA in a molar ratio of 1.61:1.42:2.14:6.42:2.39. QAP-2 was consisted of Rha, Ara, Man, Gal and GalA with a relative molar ratio of 2.05:1.21:1.00:1.82:1.53.Cordeiro *et al.*, has reported that polysaccharides from quinoa are rich in arabinan [13]. However, the polysaccharides purified here were inconsistent with this finding, which may be due to different purification methods.

### 2.3. Fourier Transform Infrared (FT-IR) Analysis

**Figure 2:**
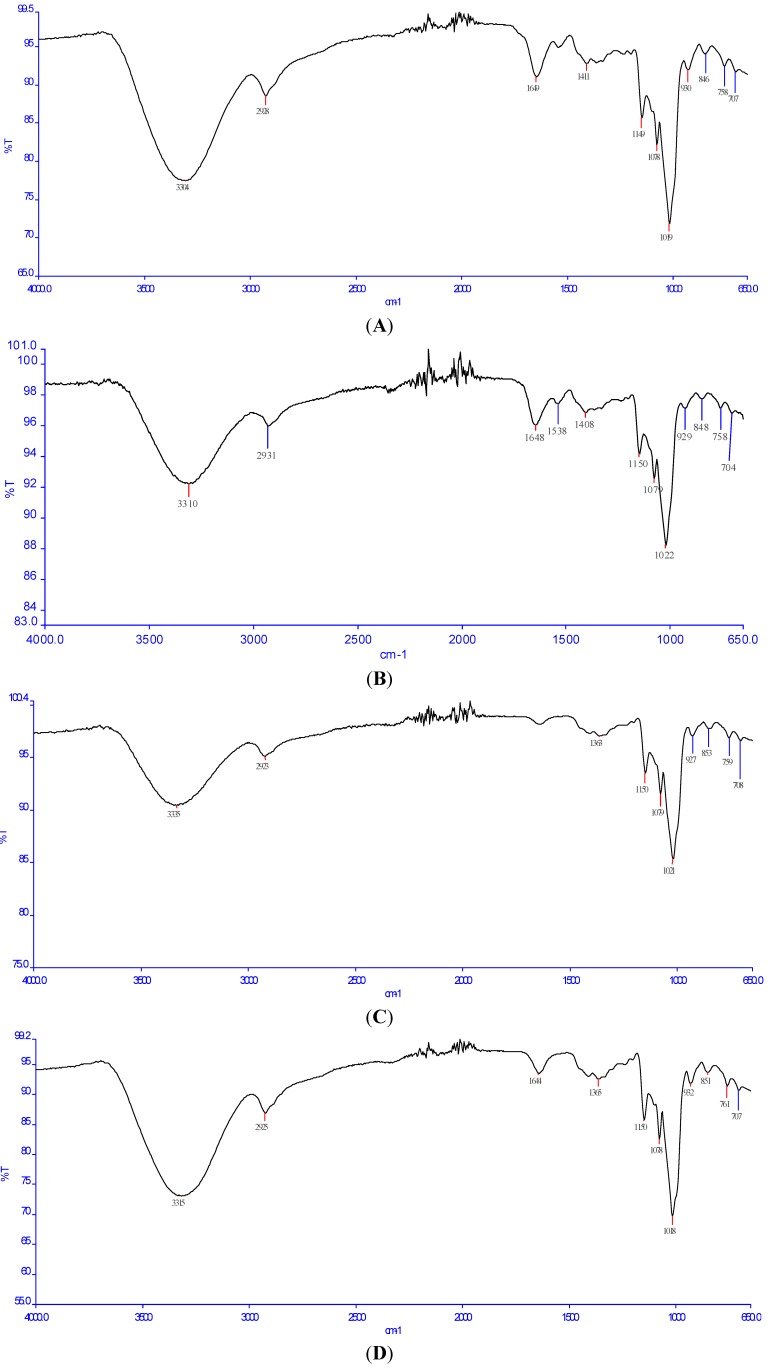
(**A**) Fourier transform infrared (FT-IR) spectrum of QWP-1; (**B**) FT-IR spectrum of QWP-2; (**C**) FT-IR spectrum of QAP-1; and (**D**) FT-IR spectrum of QAP-2.

Fourier transform infrared (FT-IR) spectroscopy is typically used for the qualitative measurement of organic functional groups. The infrared spectra of QWP-1, QWP-2, QAP-1 and QAP-2 were presented in Figure 2. The four infrared spectra displayed a broadly stretched intense peak at 3300–3500 cm^−1^ characteristic of hydroxyl groups and a weak C–H band at 2920–2980 cm^−1^. The prominent band between 1010 and 1100 cm^−1^ was representative of a pyran structure. The peak around 1650 cm^−1^ in Figure 2a,b,d were due to the bound water [14]. The absorption around 1410 cm^−1^ in Figure 2a,b was the antisymmetric and symmetric COO– stretches [15]. The absorption band from 1300 to 800 cm^−1^, called the “finger print” region, was related to conformation and surface structure of the molecule. Although these bands are hard to explain [16], peaks at 950–1200 cm^−1^ suggested the presence of C–O–C and C–OH link bonds [17].

### 2.4. Antioxidant Activities

DPPH radical and ORAC radical cation assays, expressed as tetraethylammonium chloride (TEAC) values, were utilized for the evaluation of free radical-scavenging properties of the four fractions of quinoa polysaccharide. The use of more than one method is recommended to give a comprehensive prediction of antioxidant efficacy [18]. The results are shown in Table 1. In this study, QAP-1 showed the highest DPPH value, followed by QAP-2. The relatively stable organic radical, DPPH, has been widely used in the determination of antioxidant activity of different plant extracts. Although the DPPH method is simple and rapid, it has generally a relatively small linear reaction range of only 2–3-fold. Furthermore, DPPH radical is decolorized by reducing agents as well as H transfer, which may contribute to inaccurate interpretations of antioxidant capacity [19]. ORAC is the only assay that combines both inhibition time and degree of inhibition into a single quantity. QWP-2 exhibited the strongest ORAC value. The antioxidant capacities of polysaccharides from quinoa have been reported here for the first time.

**Table 1:** DPPH and ORAC values for different polysaccharide fractions.

Varieties	DPPH	ORAC
QWP	4.76 ± 0.16 ^b^	14.62 ± 0.84 ^c^
QAP	4.24 ± 0.28 ^b^	14.66 ± 0.42 ^c^
QWP-1	4.78 ± 1.03 ^b^	27.19 ± 1.74 ^b^
QWP-2	4.94 ± 0.01 ^b^	40.90 ± 0.54 ^a^
QAP-1	6.61 ± 0.93 ^a^	29.22 ± 2.33 ^b^
QAP-2	5.78 ± 0.50 ^a^	24.86 ± 2.63 ^b^

Data were expressed as mean ± standard deviation of triplicate samples. The antioxidant activity was expressed as μmol 6-hydroxy-2,5,7,8-tetramethylchroman-2-carboxylic acid (Trolox)/gram. Values within a row followed by different letters are significantly different at *p* < 0.05. Data are expressed as mean ± SD (*n* = 3).

### 2.5. Immunomodulatory Activity Analysis

**Figure 3:**
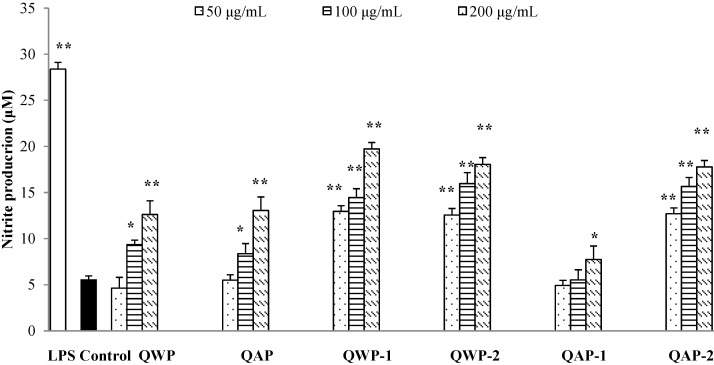
Effects of different concentrations of quinoa polysaccharides on NO production in macrophages RAW 264.7. Cells were incubated for 24 h with the indicated concentrations of polysaccharide fractions or lipopolysaccharide (LPS) (1 μg/mL). Control cells were incubated with medium alone. Values are mean ± SD (*n* = 3). * *p* < 0.05 and ** *p* < 0.01 compared to control.

Nitric oxide (NO) is an important signaling molecule that acts in many tissues to organize a diverse range of physiological process. Nitrite concentrations in the supernatant of polysaccharide stimulated macrophages were determined as a reflection of NO production. In this study, QWP-1, QWP-2, QAP-1 and QAP-2 were able to stimulate macrophages to produce NO in a dose-dependent manner (Figure 3). QWP-1 appeared to be the most potent, and induced significantly higher (*p* < 0.05) NO production at the concentration of 200 μg/mL, compared to control. QWP-1, QAP-1 and QAP-2 stimulated lower levels of NO, demonstrating their weaker effect on macrophages activation.

**Figure 4:**
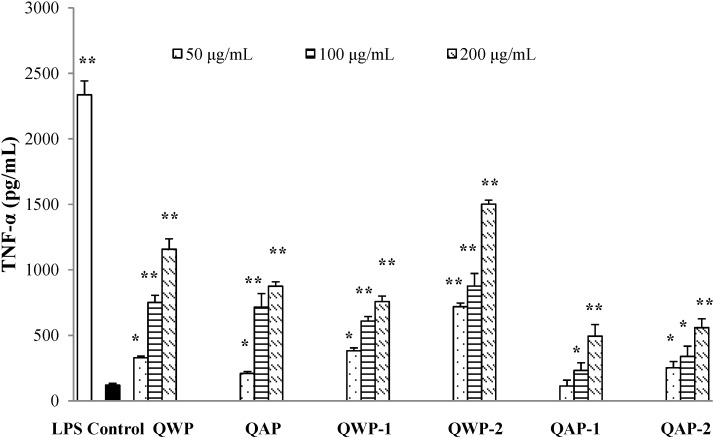
Effects of different concentrations of quinoa polysaccharides on tumor necrosis factor-α (TNF-α) production in the macrophage cell line RAW 264.7. Cells were incubated for 24 h with the indicated concentrations of polysaccharide fractions or LPS (1 μg/mL). Control cells were incubated with medium alone. Values are mean ± SD (*n* = 3). * *p* < 0.05 and ** *p* < 0.01 compared to control.

**Figure 5:**
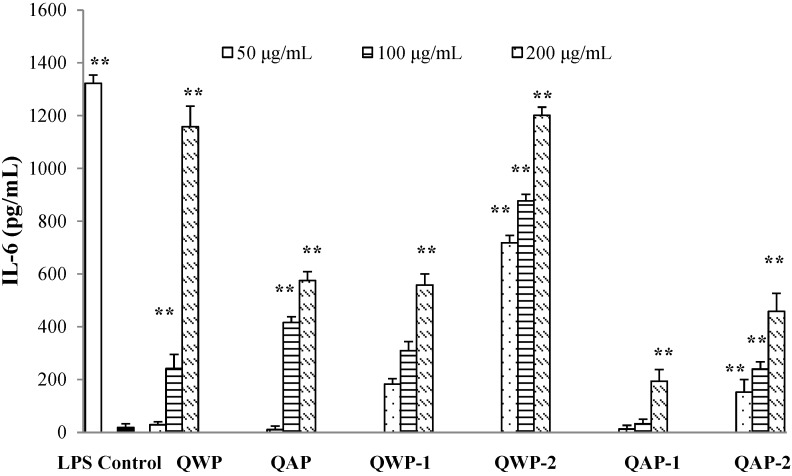
Effects of different concentrations of quinoa polysaccharides on IL-6 production in the macrophage cell line RAW 264.7. Cells were incubated for 24 h with the indicated concentrations of polysaccharide fractions or LPS (1 μg/mL). Control cells were incubated with medium alone. Values are mean ± SD (*n* = 3). ** *p* < 0.01 compared to control.

In this work, we investigated the effect of polysaccharides on the production of tumor necrosis factor-α (TNF-α) and IL-6 from RAW264.7 macrophages (Figure 4 and Figure 5). WQP-2 showed the strongest activation of TNF-α and IL-6 at the whole concentration range. The two water-extractable polysaccharide fractions had higher activities than two alkali-extractable polysaccharide fractions. It has been extensively shown that the immunomodulating activity of polysaccharides is dependent on their chemical composition, molecular weight, conformation, glycosidic linkage, and degree of branching [20]. Previously, we have observed that the saponins from quinoa seeds can prevent and control inflammation [10]. Similar results were obtained by Azike, *et al.* [21], who reported that crude polysaccharide was immuno-inhibitory, whereas saponins were immuno-stimulatory in ginseng. These extract-related anti-inflammatory and pro-inflammatory effects may be considered as the “Yin and Yang” actions of ginseng.

## 3. Materials and Methods

### 3.1. Materials and Regents

*C. quinoa* seeds were purchased from Jingle (Shanxi, China). DEAE Sepharose Fast Flow and Sephacryl S-300 High Resolution were obtained from GE Healthcare Bio-Sciences Co. (Piscataway, NJ, USA). Monosaccharide standards d-fructose and d-glucose were purchased from Sigma–Aldrich (Shanghai, China). Dulbecco’s Modified Eagle’s Medium (DMEM), Roswell Park Memorial Institute (RPMI) 1640 medium, LPS, Griess reagent, DPPH, 2,2'-azobis (2-amidinopropane) dihydrochloride (AAPH), and 6-hydroxy-2,5,7,8-tetramethylchroman-2-carboxylic acid (Trolox) were also purchased from Sigma–Aldrich. Fetal bovine serum (FBS) was obtained from GE Healthcare Bio-Sciences (Piscataway, NJ, USA). Raw murine macrophages (RAW 264.7) were purchased from the National Platform of Experimental Cell Resources for Sci-Tech (Beijing, China). All other chemicals and solvents used were of analytical grade unless otherwise specified.

### 3.2. Extraction, Separation, and Purification of Polysaccharide

Seeds of quinoa were ground into powder. The powder was passed through a 60 mesh sieve and extracted with 95% ethanol for 3 days, and distilled water at 90 °C for 4 h, twice. The water-soluble polysaccharide (QWP) was obtained. Then the water unextractable solid was washed, dried and extracted with 0.3 M NaOH solution which contained 0.3% (*w*/*w*) NaBH_4_ at room temperature for 4 h. After centrifugation at 3500× *g* for 15 min, the supernatant was collected and adjusted to neutrality with hydrochloric acid (5 M), and then concentrated, dialyzed, and deproteinated by Sevag method [22]. The supernatant containing alkali-extractable polysaccharide was precipitated with ethanol at 4 °C for 24 h and then centrifuged and lyophilized to get QAP.

QWP and QAP were purified on an ÄKTA explore 100 purification system. QWP and QAP were dissolved in distilled water, centrifuged (13,000 rpm, 20 min), and then the supernatant was loaded on a DEAE Sepharose Fast Flow column (2.6 × 100 cm^2^) equilibrated with ultrapure water. The column was first eluted with distilled water, then with a linear gradient from 0 to 2.0 M NaCl at a flow rate of 4 mL/min. Different fractions (8 mL/tube) were collected using an automatic fraction collector, then dialyzed and lyophilized. The fractions were purified further on a Sephacryl S-300 High Resolution column (1.6 × 100 cm^2^) eluted with 0.15 M NaCl at a flow rate of 0.5 mL/min to yield two main final fractions, named QWP-1, QWP-2, QAP-1 and QAP-2, respectively. The fractions obtained were combined according to the total carbohydrate content of quantified by the phenol-sulfuric acid method under 206 nm UV detection [23].

### 3.3. Analysis of Molecular Weight

The molecular weight of QWP-1, QWP-2, QAP-1 and QAP-2 were measured using a high performance size elusion chromatography coupled with multi angle laser light scattering and refractive index (HPSEC-MALLS-RID) system, which consisted of a pump (LC-20AD, Shimadzu, Kyoto, Japan), a HPSEC column (SB-805 HQ, Shodex, Kyoto, Japan), a MALLS detector (DAWN HELEOS-IIWyatt Technology, Santa Barbara, CA, USA), and an RI detector (Optilab Rex, Wyatt Technology, Santa Barbara, CA, USA).

### 3.4. Analysis of Monosaccharide Composition

GC was used for identification and quantification of monosaccharide compositions. QWP-1, QWP-2, QAP-1 and QAP-2 were hydrolyzed by trifluoroacetic acid (2 M) at 120 °C for 4 h. The released monosaccharides were converted into the trimethylsilylated derivatives and then analyzed by GC on an Agilent 6890 instrument (Agilent Technologies, Santa Clara, CA, USA) equipped with HP-5MS column (0.25 mm × 30 m × 0.25 μm) and determined by flame ionization detector (FID). The column temperatures and other parameters were set according to previous method [24].

### 3.5. Infrared Spectrum Analysis

FT-IR spectra were obtained by using a PerkinElmer FT-IR spectrometer (PerkinElmer Spectrum 400 FT-IR, Waltham, MA, USA) in the range of 500–4000 cm^−1^.

### 3.6. Antioxidant Activities Evaluation

The DPPH radical-scavenging activity was determined using the method reported by Yao and Ren [25]. DPPH (100 μM) was dissolved in 96% ethanol. The DPPH solution (1 mL) was mixed with 1 mL of the samples. The mixture was shaken and allowed to stand at room temperature in the dark for 10 min. The decrease in absorbance of the resulting solution was monitored at 517 nm after 10 min. The results were expressed in μM of Trolox equivalents (TE) per gram. All determinations were performed in triplicates.

The determination of ORAC activity was determined as described previously with some slight modification [26]. Fifty microliter of the sample at different concentrations (0–5 mg/mL) was mixed with 50 μL fluorescein solution in a 96-well microplate, and then 150 μL of AAPH were added to each well rapidly. To build the blank decay curve and Trolox standard decay curve, 50 μL blank (methanol) or Trolox standard solution were added instead of the sample solution. The microplate was immediately placed into the Synergy™ microplate fluorescence reader (Bio-Tek Instruments, Inc., Winooski, VT, USA), and recorded every minute for 80 min. Fluorescence filters of the plate reader were set at 485 nm with a tolerance of ±20 nm for the excitation wavelength and set at 530 nm with a tolerance of ±20 nm for the emission wavelength. The temperature of the plate reader was set at 37 °C. Each ORAC value of the samples was calculated by using a regression equation between the Trolox concentration and the net area under the fluorescence decay curve (AUC). The net AUC corresponding to a sample was calculated by subtracting the AUC corresponding to the blank. ORAC values were expressed as μM Trolox equivalent (TE) per gram. All determinations were performed in triplicates.

### 3.7. Assay for Immunomodulatory Activity

Cells were incubated in medium alone (control group) or medium containing various concentrations of polysaccharides fractions (1, 10, 50, 100, 150 μg/mL) or lipopolysaccharide (LPS, 1 μg/mL) as a positive control. Cells were incubated at 37 °C in 5% CO_2_ for 24 h, and then the supernatants (50 μL) were pipetted from the medium and mixed with an equal volume of Griess reagent. After incubation for 15 min at room temperature, the absorbance was measured at 540 nm in an ELISA reader (Rayto RT-6000, Shenzhen, China). The concentration of nitrite was calculated with reference to a standard curve obtained with NaNO_2_ (0–100 μM).

RAW 264.7 cells were cultured at a density of 2 × 10^5^ cells/well for 24 h with polysaccharide fractions (10, 50, 150 μg/mL) or LPS (1 μg/mL), the control group was treated with medium alone. The supernatants were collected for the detection of tumor necrosis factor-α (TNF-α) and interleukin-6 (IL-6) production using commercial ELISA kit (BD Biosciences Pharmingen, San Diego, CA, USA) according to the instructions of the kit. The absorbance was measured at 450 and 570 nm in an ELISA reader. Cytokine quantities in the samples were calculated from standard curves of recombinant cytokines using a regression linear method.

### 3.8. Statistics

Data, which were expressed as the mean ± SD, included at least three replicates per sample. ANOVA and Tukey’s test were performed using SPSS (Statistical Package for the Social Sciences) version 17.0 (SPSS Institute, IBM, Chicago, IL, USA). All graphical representations were performed using Sigmaplot version 11.0 (SPSS). Statistical significance was set at *p* < 0.05.

## 4. Conclusions

In this study, we obtained two water-extractable polysaccharide fractions and two alkali-extractable polysaccharide fractions from the seeds of quinoa by DEAE Sepharose Fast Flow and Sephacryl S-300 HR column chromatography. Results indicated that the QAP-1 and QWP-2 showed the highest antioxidant activity in the DPPH and ORAC assay, respectively. QWP-2 could significantly increase NO, TNF-α and IL-6 release from macrophages. Moreover, the two water-extractable polysaccharide fractions contained higher activities than the two alkali-extractable polysaccharide fractions. Quinoa polysaccharides were found to be excellent dietary sources of natural antioxidants for health promotion; meanwhile, these polysaccharides exhibited great potential to be developed into functional foods or nutraceutical ingredients for modification oft he immune system.
